# A Comprehensive Review of Four Clinical Practice Guidelines of Acromegaly

**DOI:** 10.7759/cureus.28722

**Published:** 2022-09-03

**Authors:** Oboseh J Ogedegbe, Asfand Yar Cheema, Muhammad Ali Khan, Syeda Zeenat S Junaid, Jolomi K Erebo, Ewuradjoa Ayirebi-Acquah, Jennifer Okpara, Daramfon Bofah, Jennifer G Okon, Mishaal Munir, Gabriel Alugba, Aaron Ezekiel, Ohikhuare Okun, Tioluwani K Ojo, Eunice O Mejulu, Abdulmalik Jimoh

**Affiliations:** 1 Internal Medicine, Lifeway Medical Center, Abuja, NGA; 2 Medicine, Services Hospital Lahore, Lahore, PAK; 3 Internal Medicine, Lahore Medical & Dental College, Lahore, PAK; 4 General Surgery, Ibn-e-Siena Hospital and Research Center, Multan, PAK; 5 Internal Medicine, Shaikh Khalifa Bin Zayed Al Nahyan Medical and Dental College, Lahore, PAK; 6 Internal Medicine, Aga Khan University Hospital, Karachi, PAK; 7 Internal Medicine, General Hospital Nasarawa, Nasarawa, NGA; 8 Internal Medicine, Lekma Hospital, Accra, GHA; 9 Medicine, Windsor University School of Medicine, Cayon, KNA; 10 Medicine and Surgery, Avalon University School of Medicine, Willemstad, CUW; 11 Internal Medicine, All Saints University College of Medicine, Kingstown, VCT; 12 Medicine, Ghurki Trust Teaching Hospital, Lahore, PAK; 13 Internal Medicine, Delta State University, Abraka, NGA; 14 Community Medicine, Bingham University Teaching Hospital, Jos, NGA; 15 Internal Medicine, Barau Dikko Teaching Hospital, Kaduna, NGA; 16 Internal Medicine, Maviscope Hospital and Fertility Centre, Benin, NGA; 17 Internal Medicine, St. Nicholas Hospital, Lagos, NGA; 18 Medical School, Western Illinois University, Macomb, USA; 19 Internal Medicine, Mount Horeb Clinic and Dialysis Center, Warri, NGA

**Keywords:** treatment recommendations, insulin-like growth factor 1, growth hormone, guideline, acromegaly

## Abstract

Acromegaly is an endocrine disorder characterized by dysregulated hypersecretion of growth hormone (GH), leading to an overproduction of insulin-like growth factor 1 (IGF-1). The etiology is usually a GH-secreting pituitary adenoma with the resultant presentation of coarse facial features, frontal bossing, arthritis, prognathism (protrusion of the mandible), and impaired glucose tolerance, among others. Most pituitary adenomas arise due to sporadic mutations that lead to unregulated cellular division, subsequent tumor formation, and resultant GH hypersecretion. Major scientific organizations and authorities in endocrinology release regularly updated guidelines for diagnosing and managing acromegaly. We have holistically evaluated four data-driven and evidentiary approaches in the management of acromegaly to compare and contrast these guidelines and show their salient differences. These guidelines have been reviewed because they are major authorities in acromegaly management. In this comprehensive article, differences in the diagnosis and treatment recommendations of the discussed guidelines have been highlighted. Our findings showed that diagnosing modalities were similar among the four approaches; however, some guidelines were more specific about additional supporting investigations to confirm a diagnosis of acromegaly.

For management options, each guideline had suggestions about ideal therapeutic outcomes. Treatment options were identical but salient differences were noticed, such as the addition of combination therapy and alternative therapy in the setting of failure to respond to first and second-line treatments. Reviewing clinical guidelines for various pathologies encourages sharing ideas among medical practitioners and ensures that global best practices are adopted. Therefore, a constant review of these clinical practice guidelines is necessary to keep clinicians up to date with the latest trends in patient management.

## Introduction and background

Acromegaly is an acquired endocrine disorder characterized by dysregulated growth hormone (GH) production. The resulting excessive GH and insulin-like growth factor 1 (IGF-1) lead to physical disfigurements such as front head furrowing, enlargement of facial structures, scalp changes, hyperhidrosis, thickening of the lips, skin wrinkling, and the unrestrained growth of hands and feet [1]. Mandibular prognathism also results in dental malocclusion and a classical spacing of teeth [1]. In addition, cardiovascular, intestinal, pulmonary, and cerebrovascular complications can occur in untreated cases, leading to a 30% decrease in lifespan [2]. The estimated prevalence of acromegaly is 1:140,000 to 1:250,000. It has no gender predilection (men and women are equally affected), and the average age at diagnosis is 40 [3].

Recent studies have also shown increased prevalence and incidence of this pathology in the elderly, presumably due to increasing life expectancy [4]. Major endocrinology organizations and authorities worldwide regularly update guidelines for diagnosing and treating acromegaly. This review highlights the important similarities and differences in these guidelines to demonstrate the foremost diagnostic and treatment modalities for acromegaly patients, ensuring the best clinical outcomes.

Objectives

This article aims to evaluate four major clinical practice guidelines for acromegaly and to compare and contrast these guidelines. This will aid in an improved evidence-based approach to this pathology and ensure universal best practices are adopted in patient care.

## Review

Methodology

Our source of information was PubMed using the search terms acromegaly, management, treatment, and diagnosis in all possible combinations. In addition, four guidelines were obtained from trusted authorities on acromegaly and used for this review. They include the Endocrine Society (ES), Pituitary Society (PS), Acromegaly Consensus Group (ACG), and the American Association of Clinical Endocrinologists (AACE). We have compared information on these guidelines’ diagnosis, management, and treatment modalities, reporting the major similarities and differences.

Definition and epidemiology

Acromegaly is an uncommon hormonal disorder characterized by excess secretion of GH by a pituitary adenoma, subsequently leading to tissue overgrowth [5]. Considering acromegaly is a rare disease, extensive research is required to generate reliable epidemiological data. Most studies have recorded a prevalence between 2.8 and 13.7 cases per 100,000 persons [6,7] and an incidence between 0.2 and 1.1 cases per 100,000 persons [7]. According to a systematic review and meta-analysis in 2021, the pooled prevalence was 5.9 per 100,000 persons, while the incidence rate was 0.38 cases per 100,000 person-year [8]. The median age at diagnosis is around the fifth decade of life, which ranges between 40.5 and 47 years [9].

Pathophysiology

Acromegaly, in more than 95% of cases, is caused by a benign GH-producing pituitary adenoma [10]. However, exceedingly rare cases of malignant pituitary tumors are documented in the literature [10]. Some other etiologies include hypothalamic tumors (gangliocytoma, hamartoma, and glioma) and ectopic neuroendocrine tumors (pancreatic and bronchial carcinoid tumors), which stimulate excess secretion of GH by the pituitary somatotrophs [3,11]. In addition, acromegaly is also associated with some disorders such as the Carney complex (CNC), McCune-Albright syndrome (MAS), and multiple endocrine neoplasia (MEN) type 1 and 4 [3,11].

The dysregulation of the somatotrophs occurs as a result of uncontrolled cell proliferation associated with cell-cycle dysfunction and altered endocrine and/or paracrine factors regulating GH synthesis, GH secretion, and somatotroph cell growth [2]. A host of mutated genes have also been implicated as the cause of acromegaly, *AIP* in familial isolated pituitary adenoma [11,12], *SDH* subunits in pituitary adenomas in association with pheochromocytomas/paragangliomas (3Pa) [13,14], mutated *MAX* gene, and overexpression of *GPR101* [12]. IGF-1 is mainly produced in the liver as a result of excessive GH secretion [12]. It mediates the anabolic effects seen in acromegaly [15], which is inhibited indirectly by somatostatin stimulation and directly by somatotroph GH production [16,17].

Diagnosis

Early diagnosis of acromegaly equates to decreased morbidity and mortality and improved quality of life [18]. Therefore, all major societies and consensus groups emphasize the importance of detailed patient history and examination preceding any laboratory tests in a case of suspected acromegaly. Biochemical investigations and radiology are the primary modalities through which a diagnosis can be achieved. For the former, lack of assay standardizations (such as chemiluminescent immunometric assay or two-step non-extraction enzyme-linked immunoassay) or adequate normative data and variation of results with physiological factors may lead to discrepancies between lab results.

When acromegaly is suspected in patients with classical clinical signs and symptoms, especially acral and facial features [13], the ES guidelines recommend measuring IGF-1 levels [13].

However, some patients may present with less typical features such as sleep apnea syndrome, type 2 diabetes mellitus, debilitating arthritis, carpal tunnel syndrome, hyperhidrosis, and hypertension [13]. Clinicians must measure serum IGF-1 levels as the best next step. In patients with a pituitary mass, screening for acromegaly may be warranted. ES does not support checking random GH levels for diagnosis due to their unpredicted variability [13]. An oral glucose tolerance test (OGTT) test follows elevated or equivocal serum IGF-1 levels in the laboratory to confirm the diagnosis. If two hours after the administration of an oral glucose load and inducing hyperglycemia, the GH levels fail to fall below <1 μg/L, the diagnosis of acromegaly is made [13].

Once the biochemical diagnosis of acromegaly is achieved, pituitary imaging studies via magnetic resonance imaging (MRI) or computed tomography (CT) (if MRI is unavailable) are recommended. If a GH-secreting pituitary adenoma is found, then its size and relation to other structures in the brain must be assessed. If the pituitary gland is small, hypoplastic, or normal, an extra-pituitary source of a GH-secreting tumor must be investigated [13,19].

AACE guideline emphasizes that acromegaly is a multifaceted clinical syndrome whose presenting features can be typical or atypical, depending on the stage of disease progression [15]. Acromegaly should be suspected and diagnostic tests pursued if patients present with two or more of the following comorbidities: new-onset diabetes, diffuse arthralgias, new-onset or difficult-to-control hypertension, cardiac disease, including biventricular hypertrophy and diastolic or systolic dysfunction, fatigue, headaches, carpal tunnel syndrome, sleep apnea syndrome, diaphoresis, loss of vision, colon polyps, and progressive jaw malocclusion [14]. Serum IGF-1 levels are deemed to be the best initial test of choice because it serves as a proxy indicator of GH levels. However, unlike ES guidelines, they do not dismiss the highly variable serum GH levels as the initial test but advise its judicious use only in unique individual clinical contexts. Raised IGF-1 levels must be followed by the measurement of GH levels. A value <1 ng/mL after an OGTT (75 g of glucose orally followed by GH measurements every 30 minutes for 120 minutes) is considered normal [14,20]. Serum GH nadir after glucose administration could also be lowered to 0.4 ng/mL to increase the sensitivity of testing [14,20]. Failure to suppress GH corroborates the diagnosis of acromegaly. Additional testing of IGF-binding protein-3 or thyrotropin-releasing hormone test is not recommended because of the lack of any supporting evidence of its utility [20].

AACE also lists other tests that should be accompanied or closely followed at the time of diagnosis. They include brain imaging and visual field testing in case of optic chiasm involvement. Furthermore, assessment of anterior and posterior pituitary function and prolactin levels are also deemed advisable [14].

Of note, PS has also postulated IGF-binding protein 3 or acid-labile subunit to evaluate for inconsistent GH and IGF-1 results [21].

In summary, regardless of which guidelines are being implemented to reach the diagnosis, it is imperative to evaluate the results of the biochemical tests through the lens of clinical judiciousness. IGF-1 is correlated positively with GH and serves as a valuable indicator of the latter due to its continuous, non-pulsatile secretion, longer half-life, and less variability over the day concerning meals and exercise [20,22].

Nevertheless, it is crucial to be aware of confounding effects when interpreting the results. IGF-1 synthesis may be hampered by long-term obesity, malnutrition, and prolonged fasting in patients with and without acromegaly. Alternatively, systemic inflammation, renal failure, chronic liver disease, oral contraceptives, cirrhosis, and anorexia nervosa may cause hepatic resistance to GH, resulting in high levels of GH but modestly elevated, normal, or even low levels of IGF-1. Similarly, basal GH and nadir GH following OGTT are affected by various physiological factors, as displayed in Table 1 [20,22].

**Table 1:** Conditions affecting IGF-1 and GH levels. GH: growth hormone; IGF-1: insulin-like growth factor 1

Condition	IGF-1	Basal GH	Nadir GH
Puberty	High	High	High
Pregnancy	High	High	High
Diabetes mellitus	Low/Normal	High	High
Renal failure	Low/Normal	High	High
Liver disease	Low/Normal	High	High
Malnutrition/Anorexia	Low/Normal	High	High
Oral estrogen	Low/Normal	High	High
Critical illness	Low/Normal	High	High

Furthermore, different assays may yield different results. Therefore, laboratories must keep the same assays uniform for all patients, corrected according to gender and age, and ensure that the same assay is used from diagnosis and through the management of a specific patient. Table 2 shows the differences in diagnostic modalities [13,15,21,23].

**Table 2:** Comparison of the ES, AACE, PS, and ACG diagnostic recommendations. ES: Endocrine Society; AACE: American Association of Clinical Endocrinologists; PS: Pituitary Society; ACG: Acromegaly Consensus Group; IGF-1: insulin-like growth factor; GH: growth hormone; TRH: thyrotropin-releasing hormone; GnRH: gonadotropin-releasing hormone; OGTT: oral glucose tolerance test; MRI: magnetic resonance imaging; CT: computed tomography

Diagnostic criteria/tests	ES	AACE	PS	ACG
IGF-1	First test of choice	First test of choice	First test of choice	First test of choice
GH as an initial diagnostic test	Not recommended	To be interpreted in the clinical context	Not recommended	Not recommended
OGTT-induced GH suppression	Confirms diagnosis	Confirms diagnosis. Nadir of GH suppression <1 advised to increase sensitivity	Confirms diagnosis. Physiological factors can confound results	Confirms diagnosis (it is recommended that 75 g be used to achieve a level of standardization)
Other biochemical tests	Not mentioned.	IGF-binding protein-3 or TRH tests are explicitly named to be irrelevant	IGF-binding protein 3 or acid-labile subunit can be used to evaluate equivocal GH and IGF-1 results	TRH and GnRH stimulation tests of GH secretion have been used as a second-tier evaluation but are not recommended due to the risk of side effects
Radiological tests	MRI/CT scan of the pituitary gland	MRI/CT scan of the pituitary gland	MRI/CT scan of the pituitary gland	MRI/CT scan of the pituitary gland
Visual tests	Recommended if the optic chiasm is involved	Recommended if the optic chiasm is involved	Recommended if the optic chiasm is involved	Recommended if the optic chiasm is involved
Other tests	None recommended	Prolactin levels and pituitary function tests	None recommended	None recommended

Medical management

All guidelines agree on the evidence that the biochemical control of age-normalized IGF-1 and GH levels is the strongest predictor of medical therapy outcomes and disease control as well as morbidity and mortality. A precise and definite assessment of medical treatment outcomes is, however, a challenge due to the inconsistency of the reported assay. Despite this, studies have shown a decline in mortality over the years due to better biochemical control and more effective therapies [21].

Table 3 indicates the differences and similarities of each medical therapy option in light of the four guidelines [13,15,21,23].

**Table 3:** Comparison of ES, AACE, PS, and ACG guidelines for medical management. ES: Endocrine Society; AACE: American Association of Clinical Endocrinologists; PS: Pituitary Society; ACG: Acromegaly Consensus Group; SRL: somatostatin receptor ligands; SSA: somatostatin analogs; GH: growth hormone; IGF-1: insulin-like growth factor 1; HQ: high-quality evidence; MQ: medium-quality evidence; SR: systematic review; LAR: long-acting release

Treatment	ES	AACE	PS	ACG
SRL/SSA	Recommended as first line. SRL is used as primary therapy in a patient who cannot be cured by surgery, has extensive cavernous sinus invasion, does not have chiasmal compression, or is a poor surgical candidate	Recommended as first line. SSAs are effective in normalizing IGF-1 and GH levels in approximately 55% of patients. SSAs reduce pituitary tumor size modestly in about 25% to 70% of patients, depending on whether they are used as adjuvant or de novo therapy, respectively. The short-acting subcutaneously administered SSA octreotide is effective, especially when low cost and rapid onset of action is the goal	Recommended as first line. Extended-dosing intervals (>4 weeks) for 120 mg lanreotide may be effective among selected patients previously controlled with long-acting SRLs. Older age, female sex, lower IGF-1 levels, and tumor T2 MRI hypointensity at baseline predict more favorable long-term biochemical responses to primary lanreotide 120 mg therapy every 4 weeks (MQ, SR). They recommend that pasireotide LAR is an effective alternative for patients who did not receive much benefit from lanreotide or octreotide LAR. For patients who have shown complete or partial biochemical response on injectable octreotide or lanreotide, oral octreotides are suitable (HQ, SR)	Recommended as first line. For patients who are on SRL therapy, tumor shrinkage was observed in up to 80% of subjects. Tumor shrinkage did not show any link to biochemical remission (MQ). Response to SRL therapy was more pronounced after surgical debulking (MQ)
Pegvisomant	It is also recommended as first line	It is recommended as second line. Pegvisomant is often used in patients who respond poorly or are unable to tolerate SSAs. It is extremely effective in normalizing IGF-1 values (>90%). This includes patients who are partially or entirely resistant to other therapies	It is recommended as second line. Studies have shown a 73% biochemical control rate. For patients who are diabetic, it improves glucose metabolism independent of IGF-1 control but does not have the same effects in patients without diabetes (MQ)	It is recommended as second line in patients with persistently elevated IGF-1 levels after high doses of SRLs
Dopamine agonists	Cabergoline is first line in patients with only modest elevations of serum IGF-1 and mild signs and symptoms of GH excess. In such cases, ES suggests a trial of a dopamine agonist, usually cabergoline, as the initial adjuvant medical therapy	Recommended as first line. Cabergoline has been shown to yield better clinical results than bromocriptine. Dopamine agonists are recommended as first line. because of their oral availability and cheaper price. They recommend using dopamine agonists in patients with modestly elevated serum IGF-1 levels	Not specified	They recommend using dopamine agonists as first line occasionally in patients who prefer oral formulations, have markedly elevated prolactin, or those with modestly raised GH or IGF-1 levels
Combination therapy	They recommend the addition of pegvisomant or cabergoline in a patient with inadequate response to SRLs. Combining medical therapies may improve efficacy, reduce side effects associated with an individual medication, decrease the frequency of injections and total drug dose, and potentially offer a cost benefit and improved compliance during long-term treatment	In patients with a partial response to SSA therapy, the addition of cabergoline may be useful. In patients with a partial response to SSA therapy, pegvisomant can also be considered as daily or weekly doses	Combination therapy of SRL and pegvisomant is already being used and has shown impressive results with up to 96% biochemical control rate achieved	They recommend SRL and pegvisomant combination therapy in patients with poor response to first and second-line management modalities, improve cost-effectiveness in patients who require high-dose pegvisomant monotherapy or patients with an inability to achieve biochemical control after surgery
Radiotherapy	The third line of treatment. It is recommended in the setting of a residual tumor mass following surgery and if medical treatment is unavailable, unsuccessful, or not tolerated	The third line of treatment. It is recommended as an adjunctive treatment in patients not fully responding to medical or surgical treatments	The third line of treatment. It can be used after a response to prior surgery or medical treatments	The third line of treatment; however, is occasionally used as second line. It is indicated in patients with poor tumor growth control or failure of normalization of hormone levels with surgical or medical therapy

Surgical management

Surgery is generally the primary treatment of choice in acromegaly patients, and the commonly used transsphenoidal approach has proven to be efficient and safe [13]. The primary goal of treating acromegaly is to normalize GH and IGF levels. Transsphenoidal surgery has proven to be the treatment of choice for intrasellar microadenomas, non-invasive macroadenomas without bone invasion, and cavernous sinus invasion [16]. About 75-95% of patients with normalization of IGF-1 and biochemical control are due to surgical removal of the intrasellar microadenomas [16]. After surgery, GH levels return to normal in 71% of all cases and more than 80% of microadenomas [16]. However, these figures are dramatically reduced when the tumor is invasive or greater than 4 cm [18]. Every treatment plan has its advantages and disadvantages. The optimal aim of surgical treatment is to decrease the mortality rate in the acromegaly patient population compared to that of the general population. For effective management of this disease, complete surgical removal of the tumor must be achieved. Surgical results depend on preoperative tumor size, extension, and preoperative GH levels. Surgical tumor debulking before medical therapy can be considered appropriate if a surgical cure cannot be achieved. This is done to decrease the target volume [18].

Acromegaly and comorbidities

All four clinical practice guidelines acknowledge the presence of comorbidities as a significant part of diagnosing and managing acromegaly. These comorbidities are discussed below.

Hypertension

Excess GH in circulation leads to insulin resistance, endothelial dysfunction, and increased sodium and water retention, resulting in increased plasma volume and hypertension [23]. In addition, there is a predominance of diastolic blood pressure revelation, which increases in prevalence with age [13].

Diabetes Mellitus

The etiology of diabetes in acromegaly is associated with increased circulating GH and IGF-1, leading to insulin resistance, increased gluconeogenesis, and hyperinsulinemia. Therefore, the management of diabetes secondary to acromegaly is similar to that of the general population, and metformin should be considered first-line therapy [23]. Pasireotide is, however, contraindicated in patients with uncontrolled diabetes due to the risk of developing hyperglycemia. Adding glucagon-like peptide-1 agonists or dipeptidyl peptidase inhibitors can prevent this complication [23].

Obstructive Sleep Apnea (OSA)

OSA is a common finding in acromegaly due to pharyngeal soft-tissue hypertrophy. Therefore, it is necessary to evaluate for OSA once a diagnosis of acromegaly is made [23]. This is done by formal overnight polysomnography or home overnight oximetry [15].

Vertebral Fractures

Vertebral fractures resulting from osteoporosis are a frequent consequence of acromegaly and increased bone turnover [23]. Therefore, antiresorptive therapy should be considered if osteoporosis does not resolve or improve after medical and surgical management of acromegaly [15]. Dual-energy X-ray absorptiometry is repeated every two years to monitor progress [18].

Studies have shown that acromegaly increases the risk of colonic polyps [13]. Therefore, a colonoscopy should be performed after a diagnosis of acromegaly. Patients with polyps at screening or persistently elevated IGF-1 levels should undergo a follow-up colonoscopy [13,15,23].

Hypopituitarism

This results from the mass effect of an enlarging tumor, leading to a reduction in the levels of luteinizing hormone, follicle-stimulating hormone, thyroid-stimulating hormone, oxytocin, prolactin, and anti-diuretic hormone, affecting sexual function, fertility, and bone health [23].

In addition to the above complications, the laboratory finding of hypercalcemia necessitates the evaluation of primary hyperparathyroidism and, if present, MEN 1 [15].

## Conclusions

As the management of acromegaly can be complex, there is a need to regularly update guidelines to ensure universal best practices are upheld. Minor differences in guidelines were seen in diagnosis and management. These guidelines ensure that clinicians managing acromegaly patients have access to current information on evidence-based treatments to optimize outcomes. Guidelines will continue to be authored in the future as new management modalities are discovered. The importance of holistic multidisciplinary management of acromegaly is also emphasized, considering the diverse comorbidities associated with this disease.

## Appendices

The author and co-authors played important roles to actualize this article as shown below :

Oboseh John Ogedegbe - Conceptualization, visualization, supervision, oversight and 

leadership, writing of the original draft

Asfand Yar Cheema - Conceptualization, validation, writing of the original draft, methodology

Muhammad Ali Khan - Resources, software, visualization, 

Syeda Zeenat S. Junaid - Conceptualization, validation, resources, methodology

Jolomi K. Erebo - Resource and Journal review, formal analysis, writing of the original draft 

Ewuradjoa Ayirebi-Acquah - Writing of the original draft, data creation, project administration 

Jennifer Okpara - Writing of the article, data curation, leadership

Daramfon Bofah - Writing of the article, reviewing and revising the text

Jennifer G. Okon - Project administration, editing, concept rephrasing 

Mishaal Munir - Literature search, writing, and editing

Gabriel Alugba - Editing, concept rephrasing, and editing

Aaron Ezekiel - Drafting and revising the article

Ohikhuare Okun - Concept rephrasing and maintenance of data research integrity

Tioluwani K. Ojo - Preparation, typing, editing

Eunice O. Mejulu - Methodology and drafting of conclusion

Abdulmalik Jimoh - Project administration, review, and editing
